# Integrated zwitterionic conjugated poly(carboxybetaine thiophene) as a new biomaterial platform†
†Electronic supplementary information (ESI) available: Experimental procedures, details of electrochemical and optical property studies. See DOI: 10.1039/c4sc02200a



**DOI:** 10.1039/c4sc02200a

**Published:** 2014-09-30

**Authors:** Bin Cao, Qiong Tang, Linlin Li, Chen-Jung Lee, Hua Wang, Yanqiao Zhang, Homero Castaneda, Gang Cheng

**Affiliations:** a Department of Chemical and Biomolecular Engineering , University of Akron , Akron , Ohio 44325 , USA . Email: gc@uakron.edu ; Email: homeroc@uakron.edu ; http://gozips.uakron.edu/∼gc/index.html ; Fax: +1 330-972-7250; b Department of Chemistry , University of Akron , Akron , Ohio 44325 , USA; c Department of Integrative Medical Sciences , Northeast Ohio Medical University , Rootstown , OH 44272 , USA

## Abstract

An integrated zwitterionic conjugated polymer-based biomaterial platform was designed and studied to address some of the key challenges of conjugated polymers in biomedical applications. This biomaterial platform consists of conjugated polymer backbones and multifunctional zwitterionic side chains. Zwitterionic materials gain electrical conductivity and interesting optical properties through conjugated polymer backbones, and non-biocompatible conjugated polymers obtain excellent antifouling properties, enhanced electrical conductivity, functional groups of bioconjugation and response to environmental stimuli *via* multifunctional zwitterionic side chains. This platform can potentially be adapted to a wide range of applications (*e.g.* bioelectronics, tissue engineering and biofuel cell), which require high performance conducting materials with excellent antifouling/biocompatibility at biointerfaces.

## Abstract 2

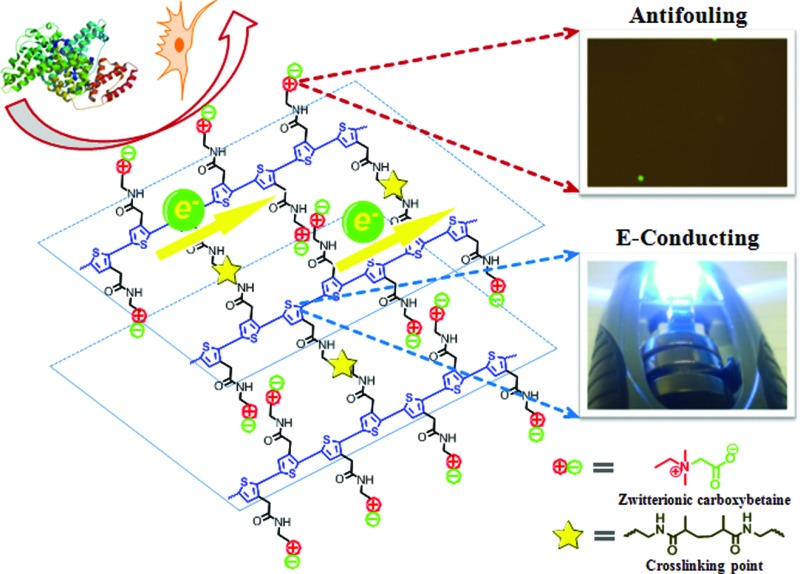
An integrated zwitterionic conjugated polymer-based biomaterial platform was designed and studied to address some of the key challenges of conjugated polymers in biomedical applications.

## Introduction

Conjugated polymers (CPs) have attracted significant interests for numerous biomedical and biotech purposes, including bioelectronics and biosensing,^1–3^ tissue engineering,^4,5^ wound healing,^6^ robotic prostheses,^7^ biofuel cell,^8^ *etc.*, due to their great design flexibility, tunable conductivity, compatible mechanical properties with soft tissues and ease of fabrication over inorganic conducting or semiconducting materials.^9^ As core components in these devices, CPs improve communications between electrochemical devices and biological systems by allowing the delivery of smaller charges or the detection of very low electrical signals, so devices can perform more efficiently.^7,10,11^ However, biomacromolecules, such as proteins and lipids, tend to adsorb on hydrophobic CPs surfaces that are originally designed for non-biological and non-aqueous systems. The nonspecific adsorption of biomacromolecules on electrochemical device surfaces reduces the sensitivity and performance of the device and triggers foreign body response that eventually leads to the failure of implanted devices.^12^ *In vivo* studies have shown that the improved electrochemical performance of devices by CP coatings could not be sustained after implantation due to the formation of non-conductive scar tissues around devices.^13–16^ Therefore, materials with tunable electronic and ionic conductivity, good biocompatibility and multi-functionality for allowing specific cell adhesion and proliferation are highly desired and urgently needed.

**Scheme 1:**
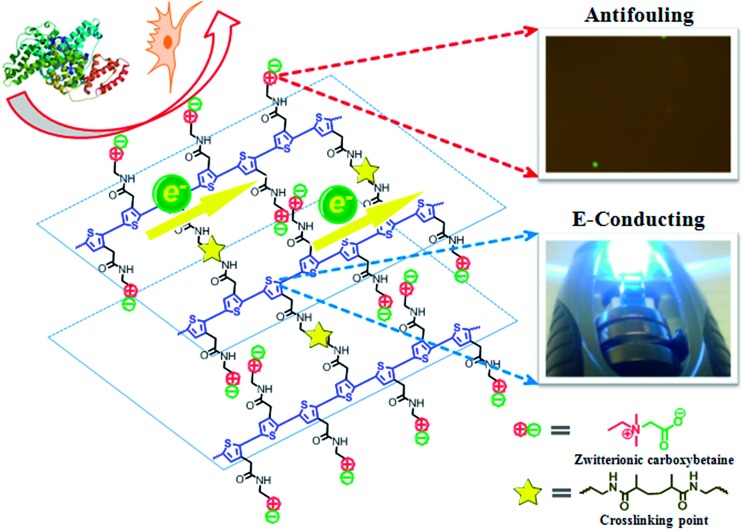
Schematic illustration of PCBTh-*co*-ThMAA hydrogel that consists of the conducting backbone and multifunctional zwitterionic side chains. Non-conducting zwitterionic materials gain electronic conductivity through the conducting backbone, and non-biocompatible CPs obtain excellent biocompatibility, enhanced electrical conductivity, sensitivity to environmental stimuli, functional groups of bioconjugation and superior antifouling properties *via* the multifunctional zwitterionic side chain.

Herein, we present a zwitterionic conjugated poly(carboxybetaine thiophene) (PCBTh) based biomaterial platform (Scheme 1) to address key challenges associated with existing CPs for biomedical applications. Conjugated polythiophene (PTh) was selected as the backbone due to its good electrical conductivity, chemical stability, low redox potential, moderate band gap and optical properties in its conducting state.^17,18^ Zwitterionic carboxybetaine (CB) was introduced as the side chains because of its excellent antifouling property,^19^ high water solubility and ionic conductivity,^20^ functionality and biocompatibility.^21^ Therefore, zwitterionic conjugated PCBTh was expected to carry highly desired properties of both zwitterionic and conjugated polymers, including superior antifouling properties, enhanced electrical conductivity and improved biocompatibility. The objective of this work is to prove the first concept of the integrated zwitterionic conjugated polymer based biomaterial platform and to lay the foundations for its further development. To test our hypothesis, we designed and synthesized a PCBTh homopolymer and a poly(carboxybetaine thiophene-*co*-thiophene-3-acetic acid) (PCBTh-*co*-ThAA) random copolymer with PCBTh as the key functional component. PCBTh-*co*-ThAA was further functionalized with methacrylamide, thiol and cell adhesion molecules and studied in the form of polymer film and hydrogel for their electrical conductivity, antifouling property to resist protein adsorption/cell attachment and functionality to incorporate cell adhesion molecules to allow the attachment of specific cells. Thus, this new platform can be readily adapted into different forms for further evaluations and different applications.

## Results and discussion

**Scheme 2:**
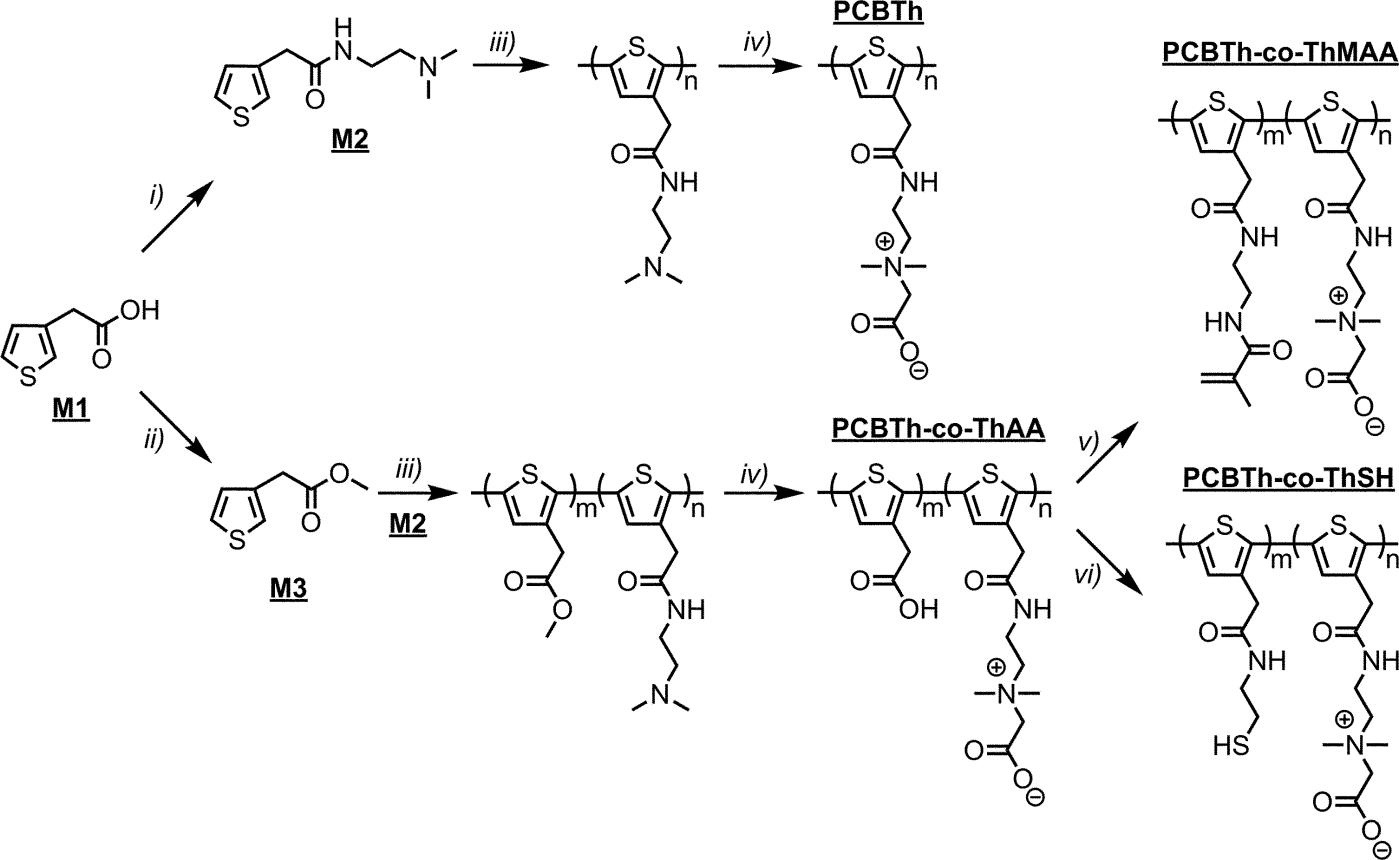
Synthetic routes to PCBTh homopolymer and its random copolymers: PCBTh-*co*-ThAA, PCBTh-*co*-ThMAA and PCBTh-*co*-ThSH. Detailed reaction conditions and procedures are included in the ESI.†

**Fig. 1:**
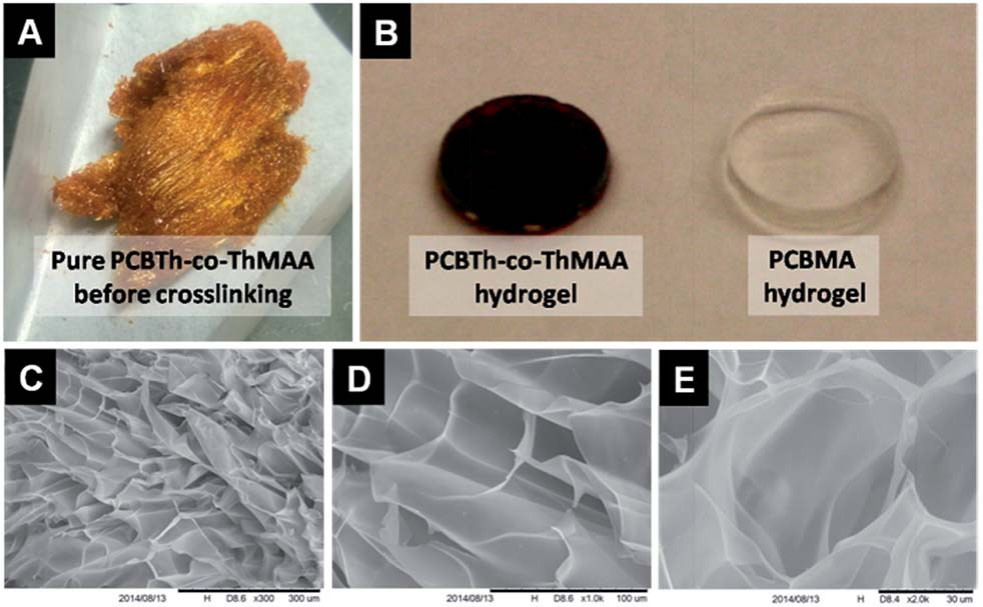
Morphological characterization of PCBTh-*co*-ThMAA in the form of (A) freeze-dried powder with metallic luster after dialysis (for hydrogel preparation), (B) wet hydrogel after equilibrated in water (with PCBMA hydrogel as a reference) (8 mm in diameter), (C–E) freeze-dried hydrogel by SEM (scale bars: (C) 300 μm, (D) 100 μm, (E) 30 μm).

Electrical conducting hydrogels, which can transport both electrons and ions, are of great interest for biomedical and biotech applications, since they provide not only a favourable electrical conducting environment but also a highest level of hydration and similarity to tissues.^22^ Since macromonomers are less toxic to cells compared to highly reactive small molecular monomers and crosslinkers for hydrogel synthesis,^23^ in this work, PCBTh homopolymer and PCBTh-*co*-ThAA random copolymer with 20 mol% ThAA repeat unit were firstly synthesized using oxidative polymerization with iron(iii) chloride in anhydrous chloroform. PCBTh-*co*-ThAA was further modified with 2-aminoethyl methacrylamide to generate crosslinkable PCBTh-*co*-ThMAA copolymer (ratio of MAA to thiophene: 10%), where pendant methacrylamide (MAA) functions as the crosslinking group (Scheme 2). PCBTh-*co*-ThMAA hydrogel was prepared with a thermal free radical initiator, VA-044 (see ESI†). The fouling control hydrogel, poly(thiophene-3-acetic acid) (PThAA),^24^ and the antifouling control hydrogel, poly(carboxybetaine methacrylate) (PCBMA),^25^ were prepared according to reported methods. The equilibrium water content of PCBTh-*co*-ThMAA hydrogel (96.3 wt%) is close to zwitterionic PCBMA hydrogel (93.7 wt%), but much higher than the control PThAA hydrogel (80.4 wt%). The porous structure of conjugated polymer hydrogel is highly favourable for electrochemical processes. Optical and scanning electron microscopy (SEM) images (Fig. 1) confirmed the desired porous structure of the PCBTh-*co*-ThMAA hydrogel.

In biological systems, electrochemical processes or devices often require embedded materials to transport both ions and electrons. It was expected that the zwitterionic conjugated PCBTh-*co*-ThMAA hydrogel could conduct electrons *via* the conjugated PTh backbone. We also hypothesized that zwitterionic side chains of PCBTh-*co*-ThMAA would enhance the overall conductivity of materials, since they can affect the self-ionization of water and subsequently improve the ionic conductivity. Electrochemical properties of PCBTh-*co*-ThMAA hydrogels in water were studied using the alternating current electrochemical impedance spectroscopy and cyclic voltammetry (CV). PCBTh-*co*-ThMAA hydrogel showed high overall electrical conductivity, which was contributed by both ionic (3.67 mS cm^–1^) and electronic (2.73 × 10^–4^ mS cm^–1^) transport. PCBTh-*co*-ThMAA hydrogel showed a good cyclicability (Fig. S9B†) and CV remained the same after 10 cycles.

Traditional biomaterials, such as poly(ethylene glycol) (PEG), zwitterionic polymers and polysaccharides, can only conduct ions instead of electrons. Conducting hydrogels, which can facilitate both electronic and ionic transport, are typically synthesized through either blending or physical crosslinking CPs with non-conducting polymeric hydrogel networks,^26,27^ however, non-conducting components can compromise electrochemical properties of conducting hydrogels.^28^ Our system avoids these problems by integrating all functional groups into one polymer chain. Electrical conductivity of undoped PCBTh-*co*-ThMAA hydrogel by electron transport was comparable to that of a doped poly(aniline) (PANI)/PEG hydrogel,^29^ which was engineered for nerve regeneration; however, the ionic electrical conductivity was improved significantly due to carboxybetaine side chains. Pan and co-workers reported that a physically crosslinked PANI hydrogel with phytic acid as the gelator and dopant could reach a much higher electrical conductivity, 0.11 S cm^–1^.^28^ Compared to their system, no dopant was added in PCBTh-*co*-ThMAA hydrogel. Electrons on conjugated polymer backbone can transfer to the reactive species, such as ions and oxygen, in biological systems, if the redox potential of the conjugated polymer is close to these species. To minimize undesired electron transfer between conjugated backbones and the aqueous environment, the CP with a proper LUMO energy level is required for each application. Higher conductivity of conjugated polymers can reduce the potential gradient of the system and the associated side reactions.

**Fig. 2:**
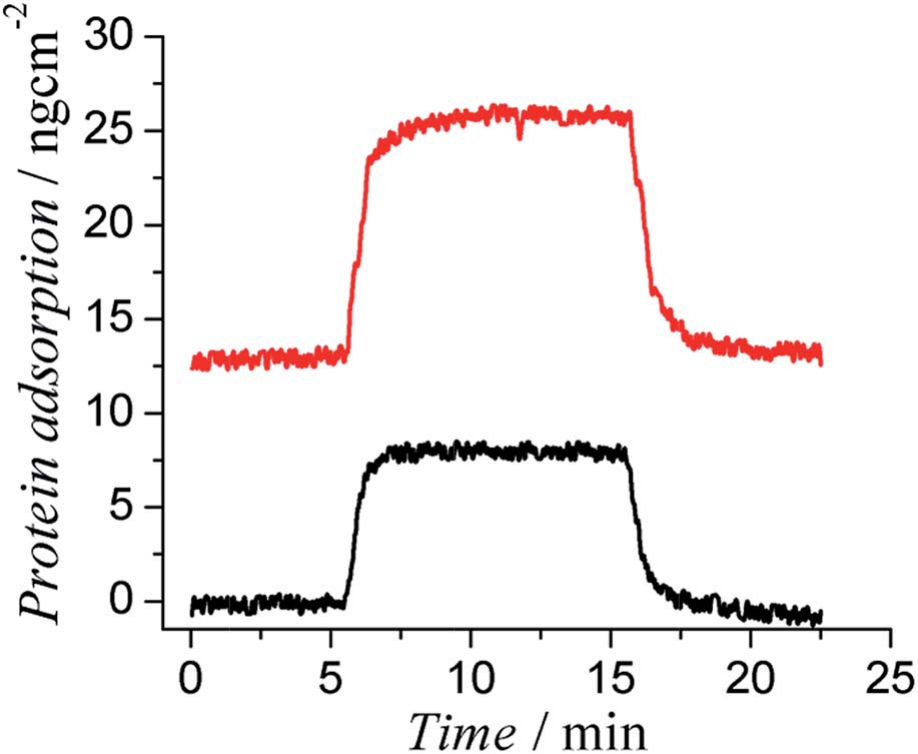
Representative SPR sensorgrams showing the very low protein adsorption of 1 mg mL^–1^ bovine serum albumin (BSA) (bottom curve) and fibrinogen (Fg) (top curve) in PBS buffer on PCBTh-*co*-ThSH modified SPR substrates.

The body responds to any foreign object by launching a series of physicochemical reactions, which are triggered by nonspecifically adsorbed proteins, and eventually forming thick and non-conductive fibrous tissues.^30,31^ Foreign body response can be attenuated if the surface of an implant can effectively resist protein adsorption and cell attachment. We hypothesized that zwitterionic side chains would endow PTh with superior antifouling properties to resist protein adsorption on their surfaces. A four channel surface plasmon resonance (SPR) sensor was used to evaluate the protein adsorption on polymer coated SPR sensor chips. Cysteamine was conjugated to PCBTh-*co*-ThAA to obtain PCBTh-*co*-ThSH. Incorporated thiol groups functioned as anchoring sites. PCBTh-*co*-ThSH was then immobilized to the surface with a “graft to” approach. Two commonly used proteins, bovine serum albumin (BSA) and human fibrinogen (Fg) that are most abundant in blood plasma, were used to evaluate the antifouling property of PCBTh-*co*-ThSH coated surfaces at a concentration of 1 mg mL^–1^. The protein adsorption on PCBTh coated gold chip surface is about 0.3 ng cm^–2^ (the detection limit of the SPR sensor) and 0.45 ng cm^–2^ for BSA and Fg, respectively (Fig. 2). In comparison, Fg adsorption on unmodified gold surface is around 300 ng cm^–2^.^32^

PCBTh-*co*-ThSH material is qualified as an ultra-low fouling material, which is defined as a surface with less than 5 ng cm^–2^ adsorbed Fg. Ultra-low fouling materials and surfaces are highly desired for biomedical applications. It was reported in a previous study that blood-contacting materials with the ultra-low fouling property would not trigger the platelet adhesion on the surface and subsequently delay the blood coagulation through contact activation pathway.^20^ Besides the surface chemistry, antifouling properties of any solid substrate are also affected by the packing density of the functional polymer coating. To rule out the effect of the surface defect and acquire their intrinsic antifouling properties to resist protein adsorption, antifouling polymers are usually studied in the form of high packing polymer brushes, which can be prepared using surface initiated polymerization techniques on various solid substrates. For example, Fg adsorption on PCBMA polymer brushes is below the detection limit of the SPR sensor (0.3 ng cm^–2^).^19^ Due to the limitation of oxidative polymerization, PCBTh-*co*-ThSH was immobilized to the surface with the “graft to” approach, which leads to a lower packing density compared to polymer brushes prepared *via* the “graft from” approach; however, the antifouling property of PCBTh-*co*-ThSH could still reach the level of the ultra-low fouling material.

**Fig. 3:**
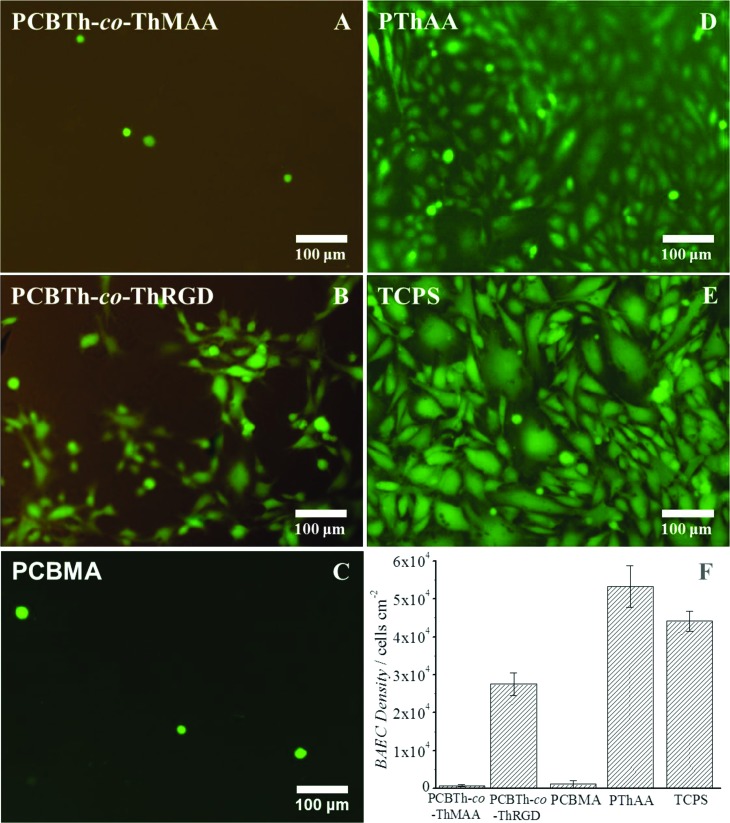
Representative fluorescence microscopy images of attached bovine aortic endothelial cells (BAECs) on (A) PCBTh-*co*-ThMAA hydrogel, (B) PCBTh-*co*-ThRGD hydrogel, (C) PCBMA hydrogel, (D) PThAA hydrogel and (E) TCPS surfaces. (F) Quantitative cell density on these surfaces.

To further evaluate antifouling properties of PCBTh at a low packing density, cell attachment studies were performed using bovine aorta endothelial cells (BAECs) on PCBTh-*co*-ThMAA hydrogel. PThAA hydrogel^24^ and tissue culture polystyrene (TCPS) were used as positive fouling control surfaces while PCBMA hydrogel was used as a positive antifouling control surface. After 24 hours' incubation, PThAA hydrogel and TCPS surfaces were almost fully covered with BAEC cells. However, there was a small amount of cells on antifouling PCBTh-*co*-ThMAA and PCBMA hydrogel surface (Fig. 3). The density of attached BAECs on PCBTh-*co*-ThMAA hydrogel surfaces was 1.5% of that on PThAA hydrogel surfaces (Table 1). To increase the solubility of the hydrophobic PTh in water, PTh needs to be modified with charged or hydrogen bond forming side chains. It is known that both positively and negatively charged surfaces will lead to protein adsorption and promote nonspecific cell attachment.^33,34^ Although hydrogels, which are based on neutral and hydrophilic polymers such as dextran, can reduce cell attachment, our previous study demonstrated that zwitterionic carboxybetaine modified dextran hydrogel can further reduce the nonspecific protein adsorption and cell attachment compared to unmodified dextran hydrogel.^35^ Zhang and co-workers demonstrated that zwitterionic PCBMA hydrogels with ultra-low fouling properties resisted *in vivo* protein capsule for at least 3 months in mice and they also observed that PCBMA hydrogels promoted angiogenesis in the surrounding tissue.^21^ We expect that zwitterionic carboxybetaine side chains of conjugated polymers will improve their biocompatibility by reducing the foreign body responses.

**Table 1:** Equilibrium water content and BAEC density on different surfaces. The percentage of the attached cells relative to PThAA hydrogel surface was calculated and presented (*n* = 3)

	PCBTh-*co*-ThMAA	PCBTh-*co*-ThRGD	PCBMA	PThAA	TCPS
% of water content	96.3	98.8	93.7	80.4	—
% of cell attachment	1.5 ± 0.5	51.7 ± 5.6	2.2 ± 1.6	100 ± 10.2	82.9 ± 5.0

While implants highly resist the nonspecific attachment of unwanted cells, they may require the attachment and proliferation of specific types of cells (such as endothelial cell, neural cell, *etc.*) on their surfaces to improve signal transduction and material integration with the biological system.^1–3^ Thus, materials at the biointerfaces are required to provide functional groups to conjugate cell adhesion molecules or other bioactive moieties in a controllable manner. It was demonstrated that a cell adhesion peptide, CRGDS, could be conveniently incorporated into PCBTh-*co*-ThMAA hydrogel *via* thiol-acrylamide Michael type reaction and the resulting PCBTh-*co*-ThRGD subsequently formed a hydrogel *via* the remaining MAA as crosslinkers by the same method for synthesizing PCBTh-*co*-ThMAA hydrogel. BAECs were expected to bind CRGDS-functionalized surfaces *via* αvβ3 integrin on their surface. As shown in Fig. 3 and Table 1, the cell density of BAEC cells on the CRGDS-functionalized copolymer (PCBTh-*co*-ThRGD) hydrogel was 51.7% and 62.3% relative to that on PThAA hydrogel and TCPS respectively. Specific cell attachment on a substrate depends on the density of the cell adhesion molecule on its surface. According to the nuclear magnetic resonance (NMR) measurement, the degree of substitution (DS) of CRGDS, which is defined as the number of the peptide chain per 100 thiophene repeat unit, is 1–2%. A recent study showed that 1% RGD peptide in an antifouling phosphorylcholine hydrogel could lead to ∼70% attachment of C2C12 and SKOV3 cells compared to TCPS.^36^ From the antifouling aspect, the low DS of bioactive molecules is desired, since excessive charged or hydrophobic bioactive molecules on a material/surface may compromise its antifouling properties and lead to the nonspecific attachment of unwanted cells. The water content of PCBTh-*co*-ThRGD hydrogel (98.8 wt%) was comparable to that of PCBTh-*co*-ThMAA hydrogel. The results indicated that cell attachment was due to the incorporation of cell adhesion molecules. In PCBTh-*co*-ThRGD hydrogel, DS of incorporated CRGDS or other cell adhesion molecules can be controlled by adjusting the ratio of CRGDS to methacrylamide according to the requirements of different applications.

**Fig. 4:**
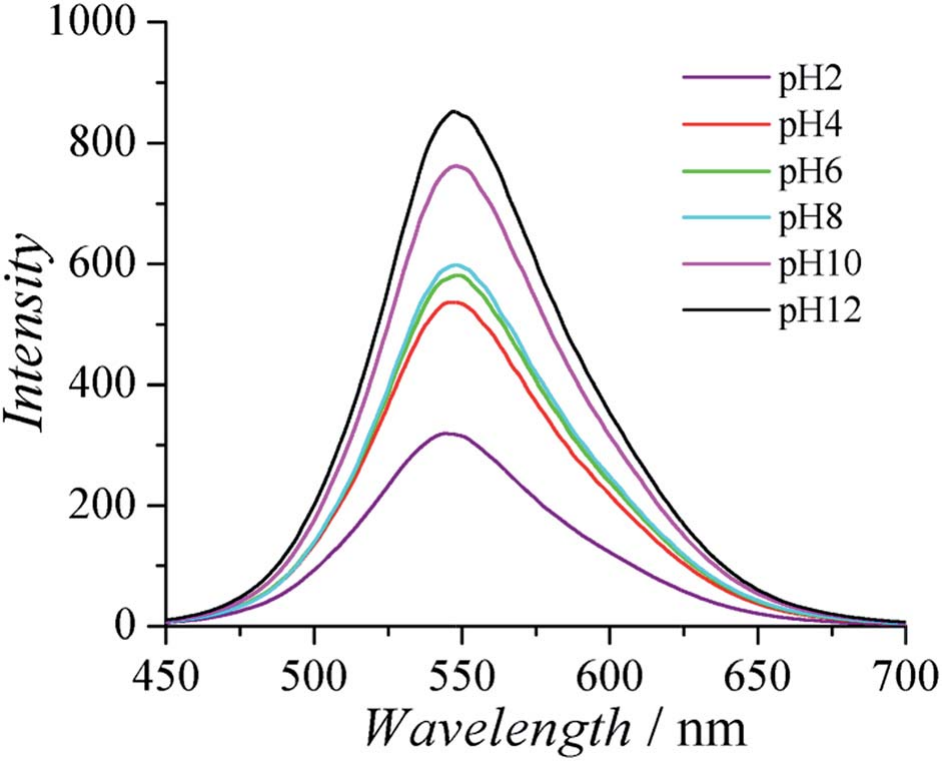
Fluorescence spectra of PCBTh in 20 mM phosphate solution at pH 2 (purple, the bottom line), 4 (red, second to the bottom line), 6 (green, third to the bottom line), 8 (cyan, third to the top line), 10 (pink, second to the top line) and 12 (black, the top line).

CPs are known to have interesting optical properties in response to environmental stimuli such as ionic strength, pH, and temperature. The optical properties of CPs are very attractive for biomedical applications, since they are complimentary to the electrical conducting properties of CPs and can provide the extra freedom for materials/devices to monitor the surrounding environment. Fluorescence spectra of PCBTh were measured in response to pH changes in 20 mM phosphate solution. It was found that the fluorescence intensity of PCBTh was very sensitive to pH changes. As shown in Fig. 4, the maximal emission signal occurred at a similar wavelength (around 548 nm) under different pH values, but fluorescence intensity changed dramatically. As the pH value of the solution changed from 12 to 2, the emission intensity of PCBTh at 548 nm gradually decreased and eventually dropped to about 30% of the original intensity. The side chain of CPs determines their overall planarization, solubility in a solvent and assembly behaviour, which subsequently affects their optical properties;^32,33^ however, the detailed mechanism(s) of optical sensitivity of PCBTh in response to pH changes is still unclear. In carboxybetaine side chains, the length of the spacer between amine and carboxylate affects the acidity of carboxylate.^37^ The p*K*_a_ of carboxylate of carboxybetaine with 1-methylene spacer is around 2.^38^ The majority of carboxylates remain at the anionic state for pH > 3 conditions. The permanently charged quaternary ammonium groups help PCBTh maintain in the soluble state at all pH conditions. Thus, the pH sensitive phenomenon of PCBTh cannot be explained by the mechanism proposed for anionic CPs in which the self-assembly of polymer chains occurs when the solubility of polymers changes dramatically under different pH conditions. A systematic study is needed to elucidate the mechanism of pH sensitivity.

The performance and lifetime of electrochemical devices are significantly influenced by interfacial mechanisms occurring at the device/biological environment interface, including biofouling,^39,40^ foreign body response,^41^ loss of structural integrity and infection.^42^ We believe that zwitterionic CPs can potentially address these challenges in various forms. Linear PCBTh can be used to modify electrode as a thin film. Crosslinked PCBTh hydrogel can be used for application that requires more stable, long term, porous, higher specific surface and/or higher hydration interfaces. Cells, enzymes and/or other macromolecules can be encapsulated into the hydrogel before or after the gel formation. To develop an initial understanding on the toxicity of PCBTh, we studied the acute cytotoxicity of PCBTh polymer at various concentrations. After 24 h, the results show that PCBTh has little effect on the proliferation of BAEC cells (Fig. S11†). Systematic chronic toxicity and *in vivo* biocompatibility tests, which are eventually needed for all implantable materials, will be planned in our future study. It was found that the carboxybetaine polymer stabilized conjugated proteins and significantly prolonged protein's activity.^43^ This property is particularly useful for biosensing, since the activity of enzymes/biomolecules is another limiting factor for the function of devices. Various carboxybetaine materials^44^ have also demonstrated superior antifouling properties of resisting microbes,^19,45^ excellent biocompatibility,^46^ as well as the capability of further functionalization for applications in biosensing^47^ and drug delivery.^25,48^ These properties make carboxybetaine polymers very useful materials to fabricate biomedical devices to prevent protein adsorption, prolong the activity of biomolecules, provide functional groups for conjugation and increase the lifetime of the device.

## Conclusions

We developed a versatile and high performance zwitterionic CP platform, which integrates all desired functions into one material. Zwitterionic CPs consist of the conjugated backbone and multifunctional zwitterionic side chains. Non-conducting zwitterionic materials gain electrical conductivity through the conjugated backbone and CPs obtain excellent biocompatibility, sensitivity to environmental stimuli and controllable antifouling properties *via* multifunctional zwitterionic side chains. Unique properties from two distinct materials (conjugated polymer and zwitterionic polymer) are integrated into one material without sacrificing any properties. Through this study, we also established a better understanding of structure–function–property relationships of zwitterionic CPs. This platform can potentially be adapted for a wide range of applications (*e.g.* bioelectronics, tissue engineering, wound healing, robotic prostheses, biofuel cell, *etc.*), which require high performance conducting materials with excellent antifouling/biocompatibility at complex biointerfaces. This zwitterionic CP platform may significantly advance the development of conjugated polymers in the field of biomedicine and biotechnology.
