# Supplementary material for: Integrated zwitterionic conjugated poly(carboxybetaine thiophene) as a new biomaterial platform
Source: Chem Sci. 2014 Sep 30;6(1):782–8. doi: 10.1039/c4sc02200a (PMC5592746; doi:10.1039/c4sc02200a)
Supplement: Supplementary file 1 [file SC-006-C4SC02200A-s001.pdf]

Electronic Supplementary Material (ESI) for Chemical Science.
This journal is © The Royal Society of Chemistry 2014

Supporting Information

Integrated Zwitterionic Conjugated Poly(carboxybetainethiophene) as a New Biomaterial Platform

Bin Cao[1], Qiong Tang[1], Linlin Li[2], Chen-Jung Lee[1], Hua Wang[1], Yanqiao Zhang[3], Homero Castaneda[1,] and Gang Cheng[1,]**

[*] Prof. G. Cheng and Prof. H. Castaneda-Lopez Corresponding-Author,

[1] Department of Chemical and Biomolecular Engineering, University of Akron, Akron, Ohio 44325 (USA)

[2] Department of Chemistry, University of Akron, Akron, Ohio 44325 (USA)

[3] Department of Integrative Medical Sciences, Northeast Ohio Medical University, Rootstown, OH 44272, (USA)

Table of Content

1. Materials and General instrumentation. S2
2. Synthesis of monomers and polymers. S2
3. Hydrogel preparation S8
4. Polymer film preparation S8
5. Electrochemical study S9
6. Protein adsorption study S10
7. Cell adhesion study S10
8. Water content measurement S10
9. Cytotoxicity study S10
10. Optical properties study S12

1. Materials and General instrumentation.

Thiophene-3-acetic acid (ThAA) was purchased from Matrix Scientific (Columbia, SC, USA). 1,1'-Carbonyldiimidazole (CDI), 1-ethyl-3-(3-dimethylaminopropyl) carbo-diimide (EDC), N-hydroxysuccinimide (NHS) and tris(2-carboxyethyl)phosphine hydrochloride (TCEP) were purchased from Chem-Impex International (Wood Dale, IL, USA). Thermo initiator 2,2'-Azobis[2-(2-imidazolin-2-yl)propane]dihydrochloride (VA-044) was purchased from Wako Chemicals USA, Inc. (Richmond, VA, USA). Anhydrous tetrahydrofuran (THF),anhydrouschloroform, methanol, dichloromethane, ethyl acetate, acetonitrile, cystaminedihydrochloride, N,N'-Dimethylethylenediamine, ethyl bromoacetate, anhydrous $FeCl_3$, sodium hydroxide, phosphate-buffered saline(PBS), human fibrinogen (Fg), bovine serum albumin (BSA), fetal bovine serum (FBS), 100X penicillin–streptomycin solution and fluorescein diacetate used as a cell viability stain were purchased from Sigma-Aldrich (St. Louis, MO, USA). All chemicals were used as received without further purification. Bovine aorta endothelial cell (BAEC) was purchased from American Type Culture Collection (Manassas, MD, USA). Dulbecco's Modified Eagle's Medium (DMEM) was purchased from Life Technologies (Carlsbad, CA, USA). Water used in all experiments was purified using a Millipore Milli-Q Direct 8 Ultrapure Water system (Billerica, MA, USA). Cellulose dialysis membrane (1k cut-off) was purchased from Spectrum Labs (Rancho Dominguez, CA, USA). The AC impedance spectrum was measured by a Solartron Model 1260 Impedance/Gain-phase Analyzer with a Model 1287 potentiostat/galvanostat (UK). The UV-vis absorption spectra of polymers were collected on a Hewlett Packard 8453 UV-vis spectrophotometer (Palo Alto, CA, USA). The fluorescence emission spectra of polymers were collected on a PerkinElmer LS 55 fluorescence spectrometer (Waltham, MA, USA). SEM images were obtained on a Hitachi TM-3000 Tabletop scanning electron microscope.

2. Synthesis of monomers and polymers.

2.1 Synthesis of monomers:

M2 (N-(2-(dimethylamino)ethyl)-2-(thiophen-3-yl)acetamide). 3-Thiopheneacetic acid (4.26 g, 30 mmol) was dissolved in 100 mL of anhydrous THF in a three-necked round bottom flask, followed by the addition of 5.88 g (36 mmol) of 1,1'-Carbonyldiimidazole (CDI). The mixture was cooled in an ice-bath (0 ^oC) and kept stirring for 20 minutes under a positive nitrogen flow. 3.28 mL of N,N'-dimethylethylenediamine (30mmol) diluted in 10 mL of anhydrous THF was added dropwise with a dropping funnel. After the complete of addition, the mixture was warmed up to room temperature and kept stirring overnight. THF

was removed with a rotary evaporator, and the product was purified with silica gel column chromatography (MeOH/CH_2Cl_2/ethyl acetate, 1/10/10 (v/v/v)). Pure product was obtained as a light yellowish liquid at 67 % yield. ^{1}H NMR (300 MHz, $CDCl_3$) δ 7.31 (m, 1H), 7.15 (s, 1H), 7.02 (d, 1H, *J*= 4.8 Hz), 6.14 (s, 1H), 3.58 (s, 2H), 3.30 (m, 2H), 2.37 (t, 2H, *J*= 6.0 Hz), 2.18 (s, 6H) (Figure s1). ^{13}C NMR (300 MHz, $CDCl_3$) δ 170.72, 135.26, 128.62, 126.38, 123.15, 57.89, 45.23, 38.29, 37.18 (Figure s2).

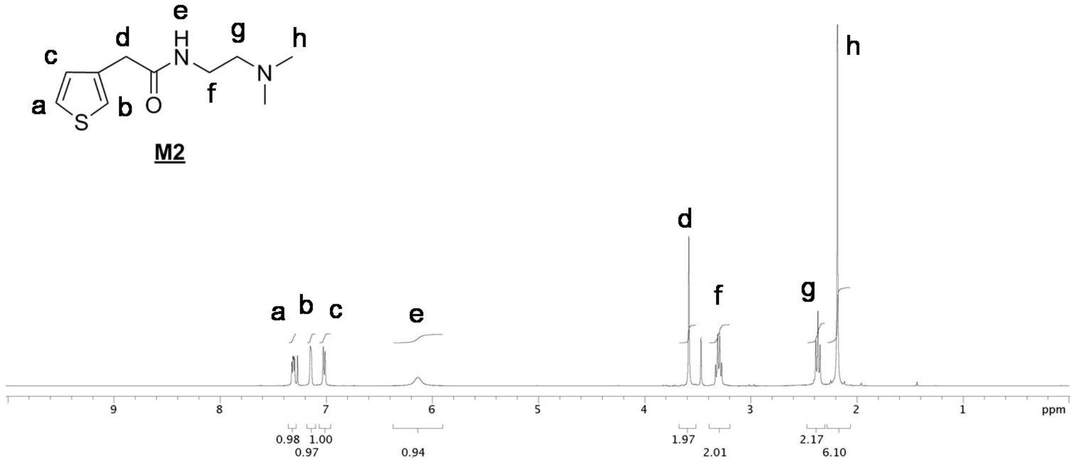

Figure S1. ^{1}H NMR of monomer M2

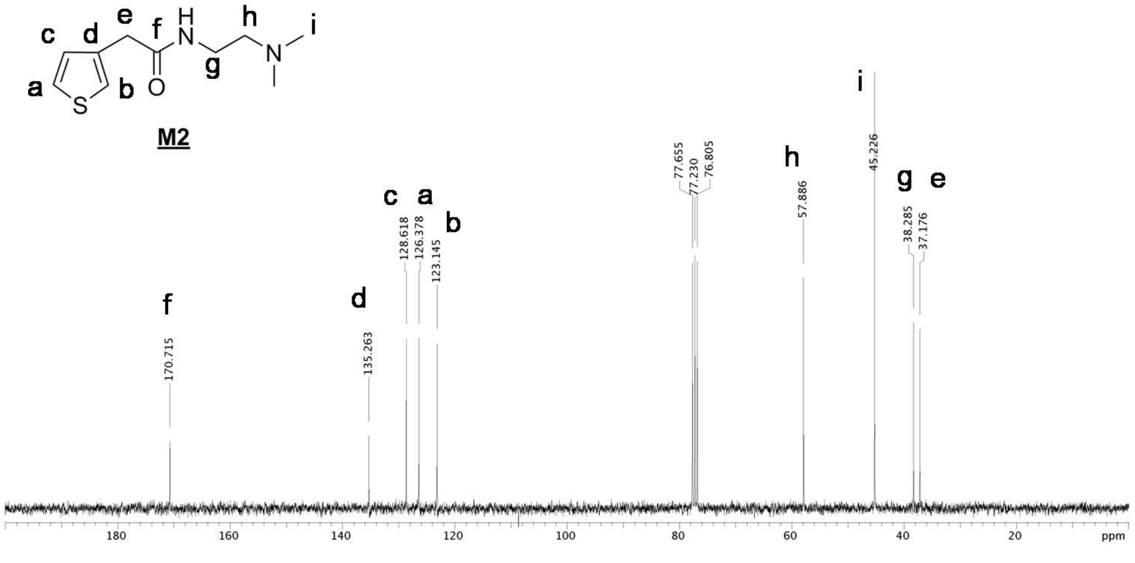

Figure S2. ^{13}C NMR of monomer M2

M3 (methyl thiophene-3-acetate) was synthesized following a previously reported method.[1] Briefly, 3-thiopheneacetic acid (8.52 g, 60 mmol) was dissolved in 50 mL of methanol with 2 drops of concentrated H_2SO_4. The mixture was heated inan oil bath and refluxed for 24 hours.

After the removal of methanol, the crude product was re-dissolved in diethyl ether, washed with DI water and dried with anhydrous magnesium sulfate. Pure product was obtained after filtration and evaporation of solvent. Structure was confirmed with ^{1}H NMR and the data was in agreement with the previous report.

2.2 Synthesis of homo-polymers and copolymers:

Homo-polymer P1. 6.11 g (37.7 mmole) of anhydrous $FeCl_3$ was suspended in 60 mL of anhydrous chloroform under a positive nitrogen flow. The mixture was cooled in an ice-bath (0 ^{o}C) and kept agitated for 30 minutes. 2.0 g (9.42 mmol) of compound 2 dissolved in 30 mL of dry chloroform was slowly added into the mixture during a period of one hour. Then the reaction was stirred for 24 hours at room temperature under nitrogen. After the reaction, the productwas washed with chloroform and dried with rotary evaporator. Then itwas re-dissolved in DI-water and purified through dialysis withcellulose dialysis membrane (1 k cut off). Water was changed daily for a week, and the solution was lyophilized to obtain **P1** at 20 % yield. ^{1}H NMR (300 MHz, D_2O) δ 6.6-7.6 (m, thiophene ring proton, 1H), 3.0-4.3 (m, thiophene ring -CH_2- and -NH-CH_2-, 4H), 2.7-3.0 (m, -CH_2-$N(CH_3)_2$, 2H), 2.0-2.7 (s, -CH_3, 6H) (Fig. S3).

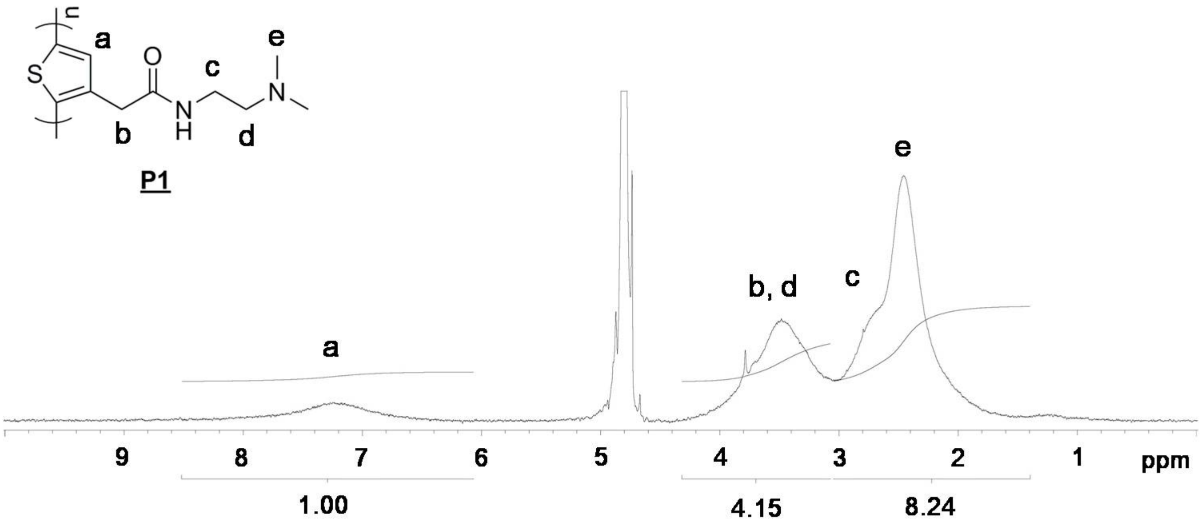

Figure S3. ^{1}H NMR of homo-polymer P1

Homo-polymer P2. 130 mg (0.6 mmole) of **P1** was dissolved in 15 mL of methanol, followed by the addition of 0.2 mL (1.8 mmole) of ethyl bromoacetate. The mixture was heated at 60 ^{o}C for 2 days under nitrogen. After concentrated with rotary evaporator, the product was precipitated in diethylether and dried under vacuum to obtain **P2** with quantitative yield. ^{1}H NMR (300 MHz, D_2O) δ 6.9-7.7 (m, thiophene ring proton, 1H), 4.2-4.6(m, $N(CH_3)_2$-CH_2-C=O and -CH_2-CH_3, 4H), 3.6-4.1 (m, thiophene ring -CH_2- and -NH-

CH_2-, 4H), 3.2-3.6 (s, -N(CH_3)$_2$, 6H), 2.9-3.1 (m, -CH$_2$-CH_2-N(CH$_3$)$_2$, 2H), 1.1-1.5(s, -CH$_2$-CH_3, 3H) (Fig. S4).

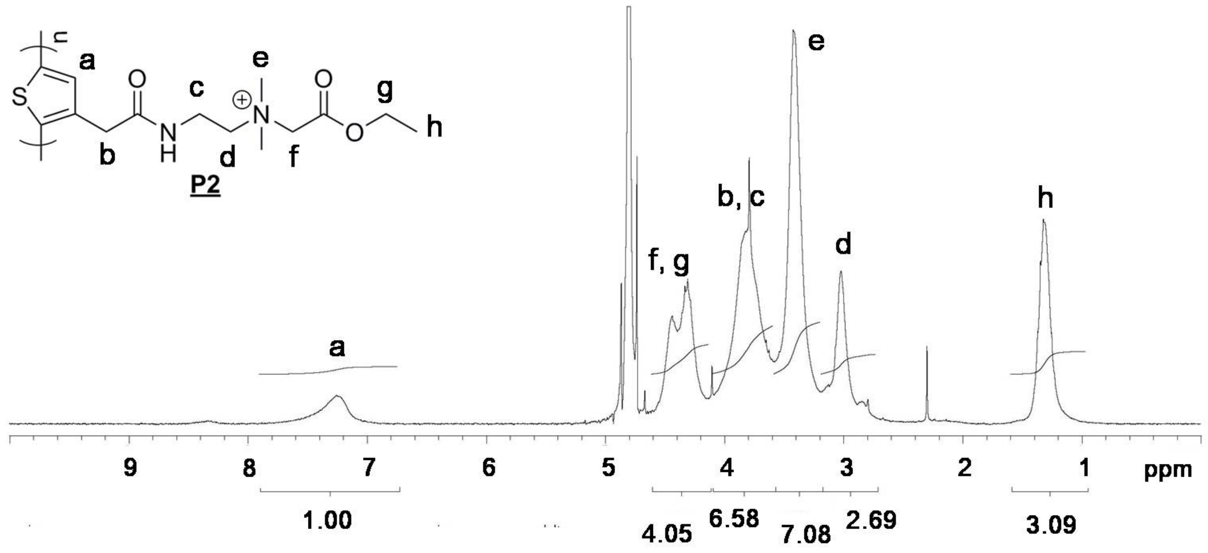

Figure S4. ^{1}H NMR of homo-polymer P2

Homo-polymer P3 (PCBTh). P2 was dissolved in DI water and passed through an ion exchange resin (Amberlite IRA-400 OH form) filled column to hydrolyze ethyl ester into the final zwitterionic form. Pure **PCBTh** was obtained as a red powder after freeze-drying with 95% yield. ^{1}H NMR (300 MHz, D$_2$O) δ 6.5-8.0 (m, thiophene ring proton, 1H), 3.5-4.5 (m, N(CH$_3$)$_2$-CH_2-C=O, thiophene ring -CH_2- and -NH-CH_2-, 6H), 3.0-3.5 (s, -N(CH_3)$_2$, 6H), 2.2-2.6 (s, -CH$_2$-CH_2-N(CH$_3$)$_2$, 2H) (Fig. S5.).

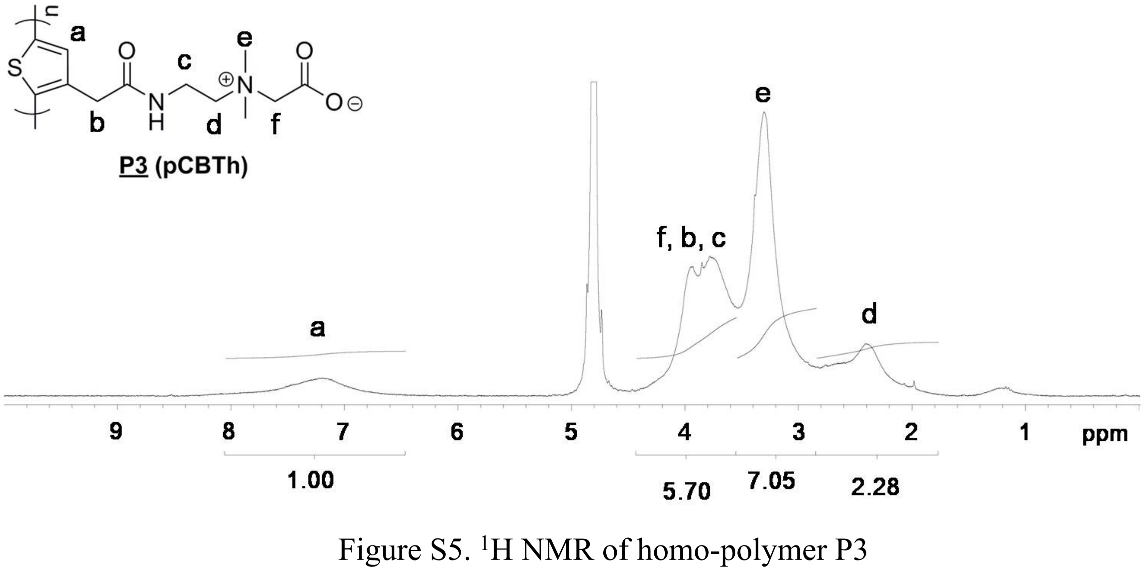

Figure S5. ^{1}H NMR of homo-polymer P3

Copolymer P7 (PCBTh-co-ThMAA) and P8 (PCBTh-co-ThSH) (Scheme 1). Monomers M2 and M3 were pre-mixed at a feeding ratio of 80:20 for the oxidative polymerization with anhydrous FeCl$_3$. The synthesis from copolymers P4 to P6 were following similar procedures to that of homopolymer **P3**. After purification from dialysis with cellulose dialysis membrane

(1 k cut off), **P6** (PCBTh-co-ThAA) was separated into two portion sand submitted to two separate reactions to synthesize **P7 (PCBTh-co-ThMAA)** and **P8 (PCBTh-co-ThSH)**.

In the first reaction, **P6** was reacted with 2-aminoethyl methacrylamide hydrochloride[2] in the presence of EDC to obtain self-crosslinkable copolymer **P7 (PCBTh-co-ThMAA)**. After dialysis, the substitution ratio of methacrylamide double bond to thiophene unit was about 10 % based on ^{1}H NMR integral values. ^{1}H NMR (300 MHz, D_2O) δ 6.6-8.0 (m, 1H), 5.5-5.8 (m, 1H), 5.2-5.5 (m, 1H), 3.5-4.5 (m, 8H), 3.0-3.5 (s, -N(CH_3)$_2$, 6H), 2.5-3.0 (m, 4H), 1.7-2.0 (s, 3H) (Fig. S6).

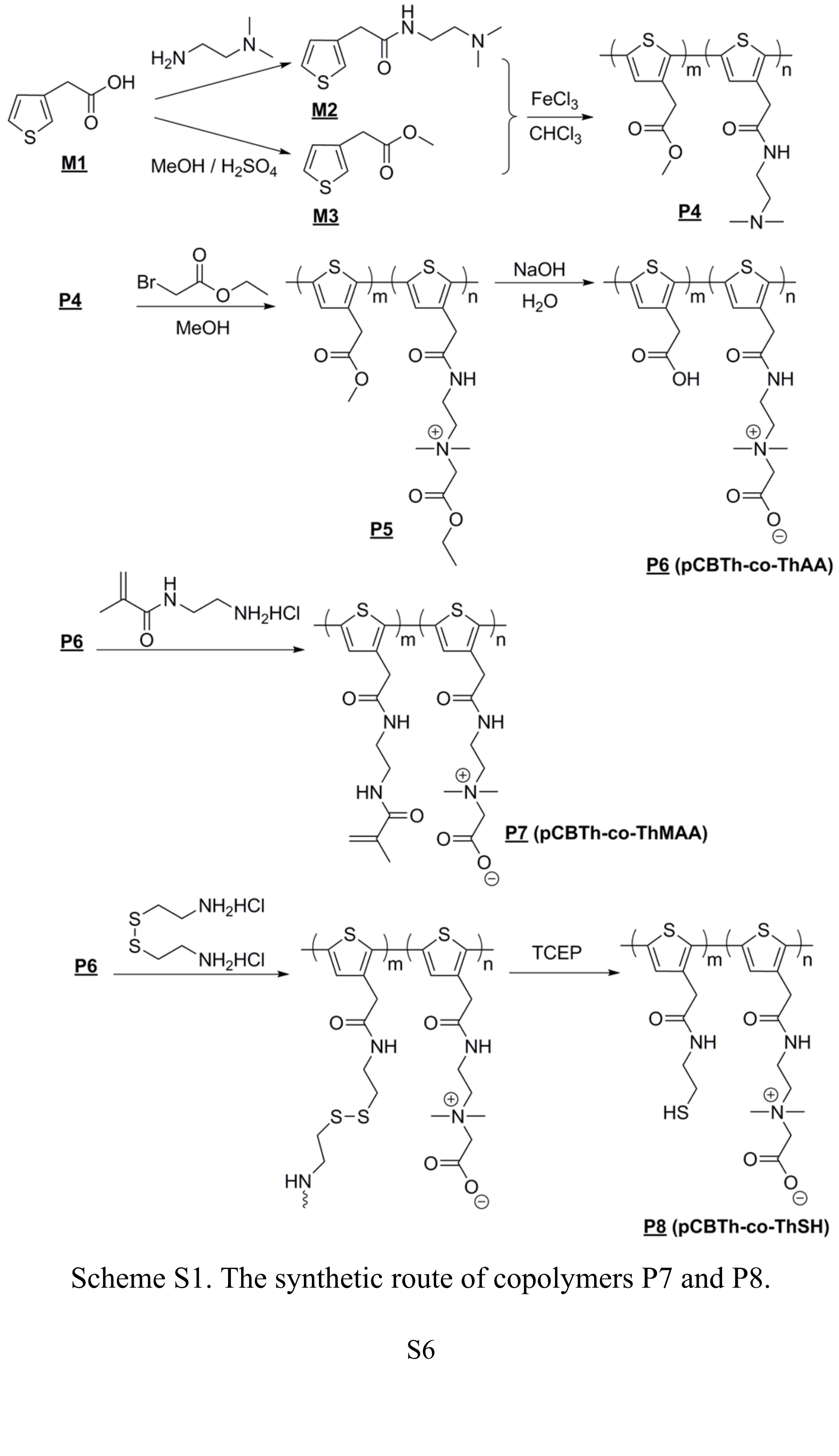

Scheme S1. The synthetic route of copolymers P7 and P8.

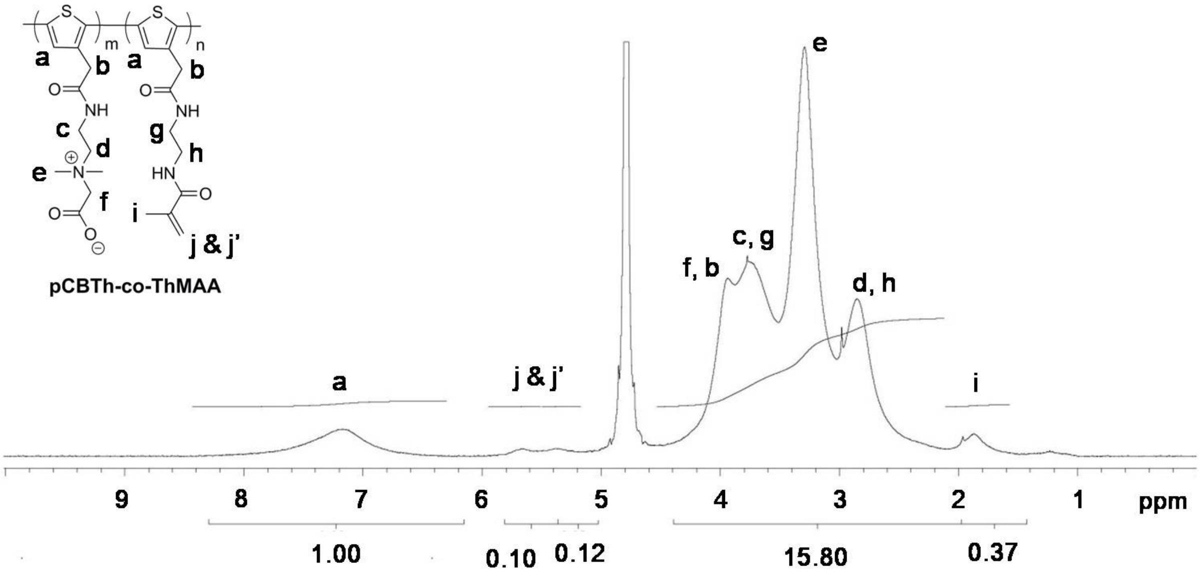

Figure S6. ^{1}H NMR of co-polymer P7 (pCBTh-co-ThMAA)

In the second reaction, **P6** was reacted with cystamine dihydrochloride using EDC/NHS chemistry,[2] followed by the reduction of disulfide with tris(2-carboxyethyl)phosphine hydrochloride (TCEP) to obtain copolymer **P8 (PCBTh-co-ThSH)**. The incorporation of free thiol groups were designed for the immobilization of copolymers **P8 (PCBTh-co-ThSH)** on gold-coated SPR sensor chips. Since the resonance from thiol (SH) containing side chain was not resolved from the overlapping signals, the actual substitution ratio of thiol groups cannot be calculated from ^{1}H NMR. ^{1}H NMR (300 MHz, D_2O) δ 6.6-8.0 (m, 1H), 3.5-4.5 (m, 8H), 2.8-3.5 (s, -N(CH_3)$_2$, 6H), 2.6-2.8 (m, 2H), 2.2-2.4 (m, 2H) (Fig. S7).

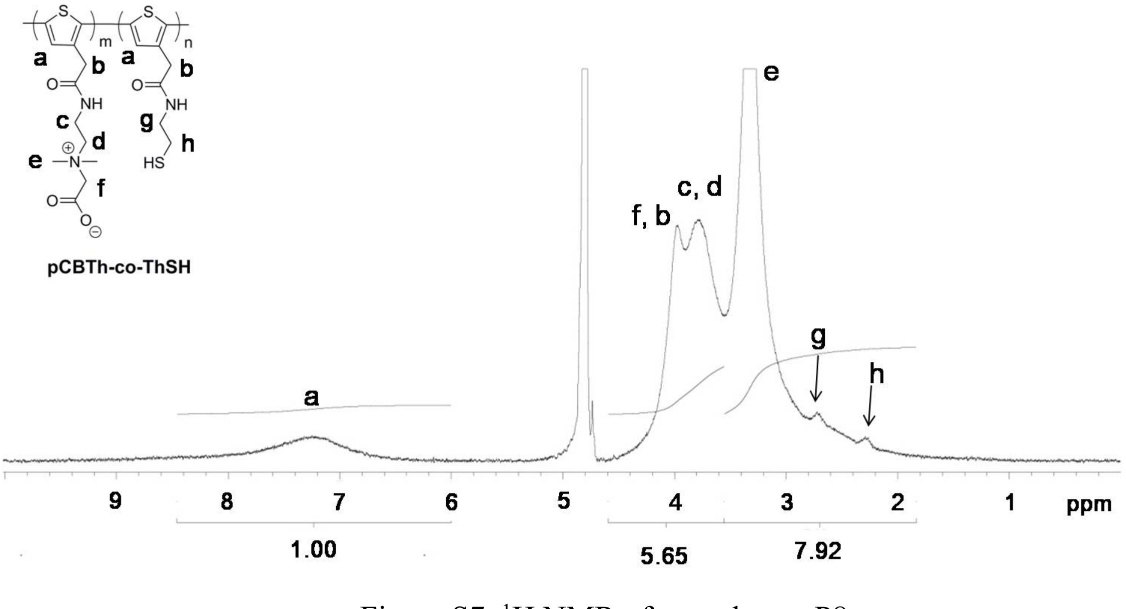

Figure S7. ^{1}H NMR of co-polymer P8

Copolymers P9 (PCBTh-co-ThRGD). A cysteine containing cell adhesion peptide, CRGDS,

was conjugated to the double bond on **P7 (PCBTh-co-ThMAA)** via the thiol-methacrylamide Michael type reaction in D_2O solution. ^1H NMR was used to monitor the reaction in real time. The ratio of double bonds to thiophene units changed from 10-12% (before conjugation) to 9% (after conjugation). So the RGD substitution ratio is estimated to be about 1-2%, equals to the final consumption of methacrylamide double bond that reflected from the NMR integral values. ^1H NMR (300 MHz, D_2O) δ 6.6-8.0 (m, 1H), 5.4-5.6 (m, 1H),5.1-5.4 (m, 1H), 3.4-4.2 (m, 8H), 2.9-3.3 (s, $-N(CH_3)_2$, 6H), 2.5-3.0 (m, 4H), 1.6-2.2 (s, 7H) (Fig. S8).

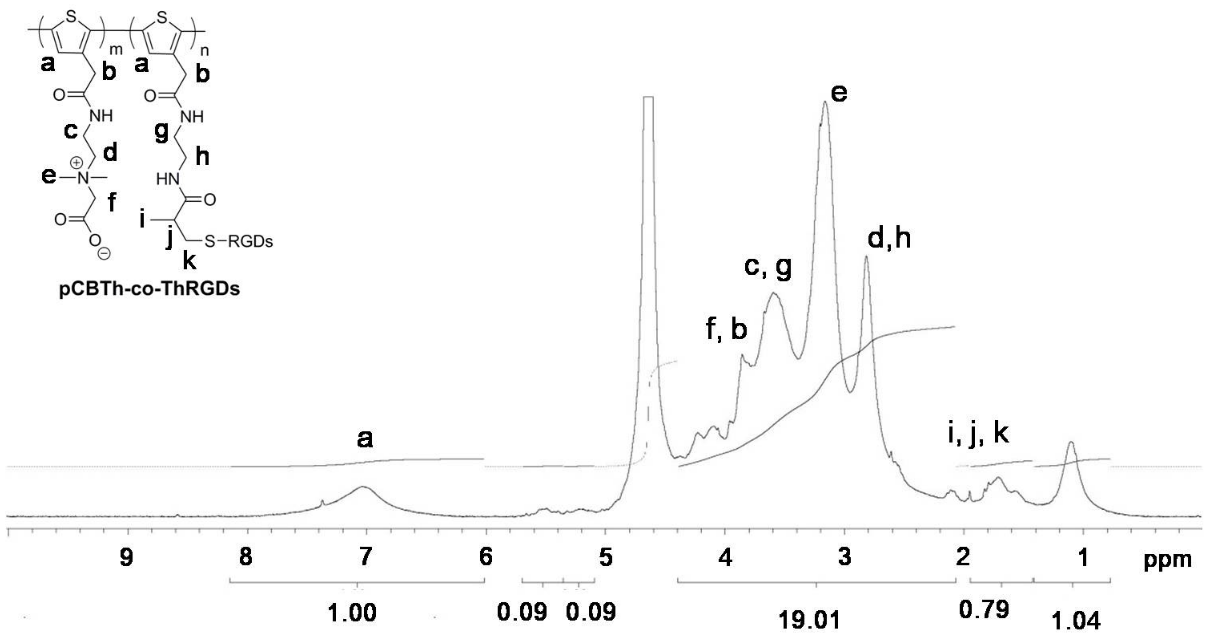

Figure S8. ^1H NMR of co-polymer pCBTh-co-RGDs

3. Hydrogel preparation

Both PCBTh-co-ThMAA and PCBTh-co-ThRGD hydrogels were prepared via similar thermo-initiated polymerizations as follows. 100 mg of copolymers was dissolved in 400 μL aqueous solution with 0.5 wt % of thermo-initiator (VA-044). Then the solution was transferred into a mold made of two quartz slides separated by a1 mm thick PTFE spacer and polymerized at 50 °C for overnight. The gel was equilibrated in DI water and water was changed daily for 7 days. The wet weight of the hydrogel samples was measured after the removal of excess water. PThAA hydrogel was prepared according to a reported method and used as a control in this study.[3]

4. Polymer film preparation

Polymer thin films were prepared with agraft-to method. Copolymer **P8 (PCBTh-co-ThSH)** with free thiol end group was prepared at the concentration of 10 mg/mL in a mixed solvent

of 90% DI-water and 10% methanol by volume. 400 μL of polymer solution was drop-casted on a gold-coated SPR chip. It was put in a petri-dish and left undisturbed until solvent evaporated at room temperature. Sample was washed with PBS and dried with filtered air before the SPR measurement.

5. Electrochemical study

The AC impedance spectrum was measured by a Solartron Model 1260 Impedance/Gain-phase Analyzer with a Model 1287 potentiostat/galvanostat in the frequency range from 0.1 mHz to 100 kHz at low amplitude voltage (~10 mV) [22]. The hydrogel sample were cut into a disc with a diameter of 6.8 mm and put between to stainless steel electrodes. The ionic and electronic conductivities of hydrogels were calculated with a previously reported method.[4] The ionic resistance, Ri, can be determined from the relationship 1/R1 = 1/Ri +1/Re, where R1 is the high-frequency semi-circle resistance from impedance data and Re is the electrical resistance measured under small applied DC potentials (-30 mV - +30 mV) using the potentiostat. Cyclic voltammetry (CV) can provide potentiodynamic electrochemical measurements and stability measurement.[5] Fig.S9 shows CV curves and the impedance curve and of PCBTh-co-ThMAA hydrogel based electrodes using a two electrode system. Fig.S9A shows the rate-dependent CVs with the potential window of 0 to 1 V at scan rates of 5, 10, 20, 30 and 50 mV/s. CV were recorded in the potential range of 0–1 V using the potentiostat.The complex diagram shows a lineal behavior at low frequencies, which indicatesthatthe mass transport is the dominant mechanism. The capacitive response at medium frequencies denotes the current carries within the material.

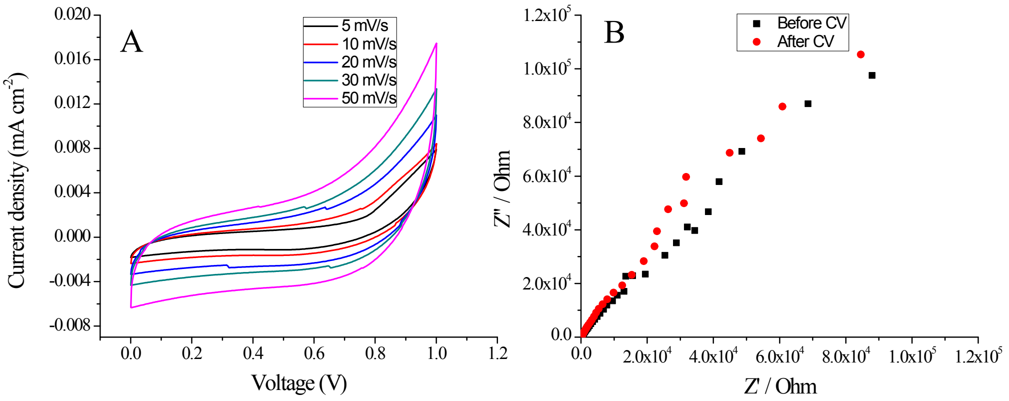

Figure S9. (A) Cyclic voltammogram curves of the PCBTh-co-ThMAA hydrogel electrode at different scan rates (5 – 50 mV·s^{-1}) and (B) Impedance curve of a PCBTh-co-ThMAA hydrogel electrode.

6. Protein adsorption study

Protein adsorption study: A custom-built four-channel SPR sensor was used to measure protein adsorption on pCBTh-co-ThSHcoated surface. Firstly, PBS solution at 50 μL min^{-1} flow rate was used to obtain a baseline signal. 1 mg $mL^{-1}$of Fg solution and1 mg $mL^{-1}$of BSA were then injected into different channels for 10 minutes followed by a PBS wash to remove any loosely bound proteins. The amount of adsorbed proteins was calculated as the change in wavelength before and after protein injection.

7.Cell adhesion study

BAECs were chosen to study cell adhesion on hydrogel surfaces, following a similar procedure as in a previous work.[6]Hydrogel samples were equilibrated in DI-water and then transferred tosterilized PBS, exposed under UV for half an hour before the experiment. BAECs were seeded on different hydrogelandcontrolsurfaces at a concentration of 10^5 cells mL^{-1} in DMEM containing 10% FBS and 1% penicillin-streptomycin, and kept in an incubator with 5% CO_2 at 37 ^oC for 24 hours. After the incubation,medium was removed from the wells and changed to the staining solution that prepared in sterilized PBS as follows. Fluorescein diacetate was dissolved at a concentration of 10 mgmL^{-1} in acetone, then50 μL of the solution was diluted in 10 mL sterilized PBS and used for stainingthe cells. After incubated for 5 min with the staining solution, surface cell coverage and cell morphology was visualized and imaged with anOlympus IX70fluorescence microscope equipped with a FITC filter at ×10 magnification.

8. Water content measurement

The water content is a basic property of hydrogel materials for biomedical applications. The wet weight of the hydrogel sample was measured after the removal of excess water. Dry weight was recorded after the samples had been freeze-dried for 48 hours. The water contents of hydrogels (Table s1) are calculated by (Wet weight – Dry weight)/Wet weight x 100%.

9. Cytotoxicity study

The cytotoxicity of the zwitterionic polymer was studied with various concentrations of pCBTh. 100 μL of BAEC cells solution, at a concentration of 10^5 cellsmL^{-1}, were incubated in a 96 well plate for 24 hours with differentconcentrations (0.5, $5x10^{-2}$, $5x10^{-3}$, $5x10^{-4}$ and$5x10^{-5}$mgmL^{-1}) of pCBTh. 6 replicates were used for each concentration. As a control, the same cells were also incubated at the same conditions without adding pCBTh. After 24 hours incubation, cells were stained with the same method as discussed in cell adhesion study. Representative fluorescence imagesof surviving cells were taken for each condition (Figure

S10), with an Olympus IX70 fluorescence microscope equipped with a FITC filter at ×10 magnification. The number of cells was counted by three replicates andrelative viability was calculated and summarized in Figure S11.

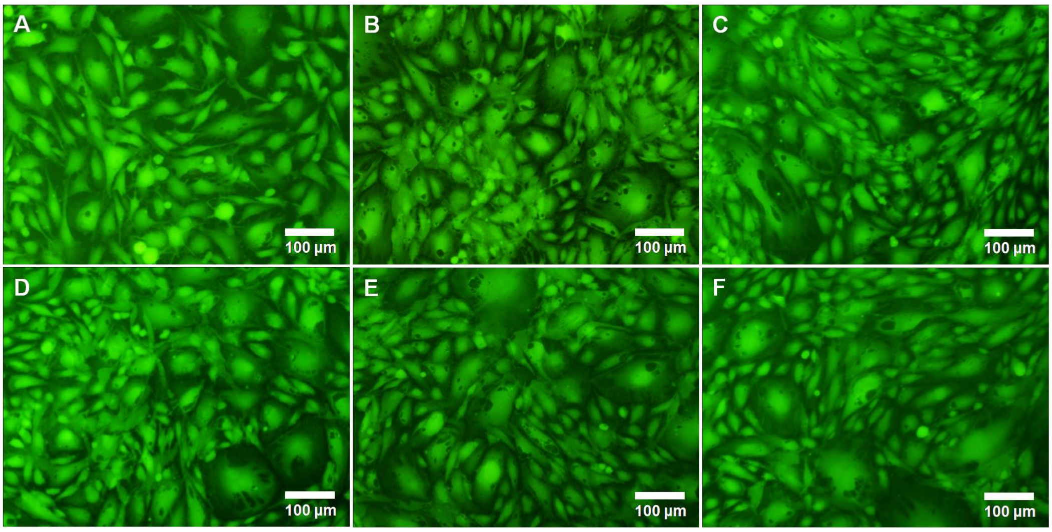

Figure S10. Representative fluorescence images of BAECs treated with a series of dilutions of pCBTh polymer A) 0.5 mgmL^{-1}, B) 5x10^{-2}mgmL^{-1}, C) 5x10^{-3}mgmL^{-1}, D) 5x10^{-4}mgmL^{-1}, E) 5x10^{-5} mgmL^{-1} and E) untreated cells, after 24 hours incubation in DMEM medium.

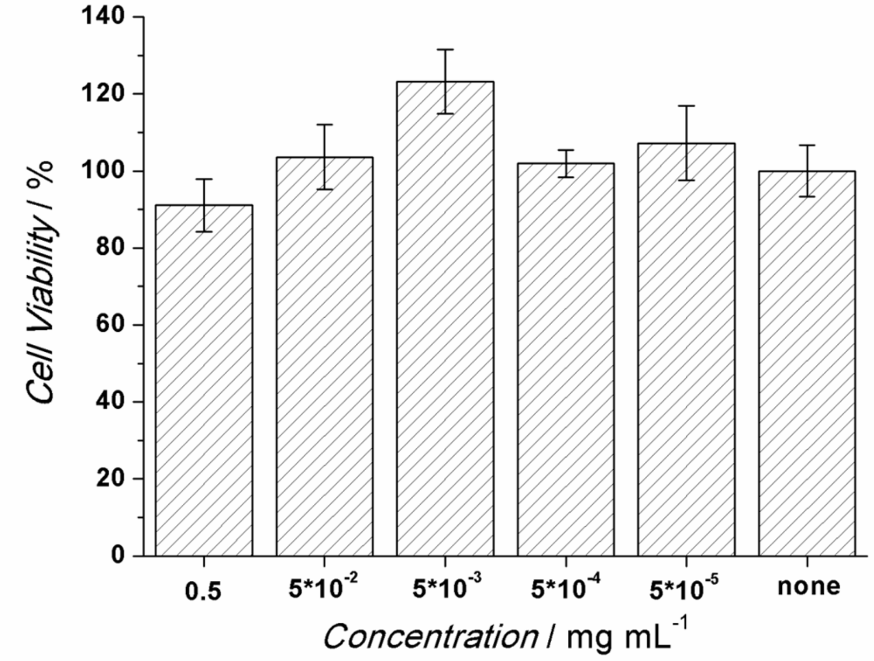

Figure S11. Representative cytotoxicity assay of BAECs treated with a series of dilutions of pCBTh polymer in culture media, expressed as a percentage of control untreated cells

10. Optical properties study

The UV-vis absorption spectra of pCBTh were collected on a Hewlett Packard 8453 UV-visspectrophotometer. Samples were prepared in 20 mM PBS buffer solution at different pH, from pH 2 to pH 12. Fluorescence emission spectra were collected on a PerkinElmer LS 55 fluorescence spectrometer, excited at 411 nm.

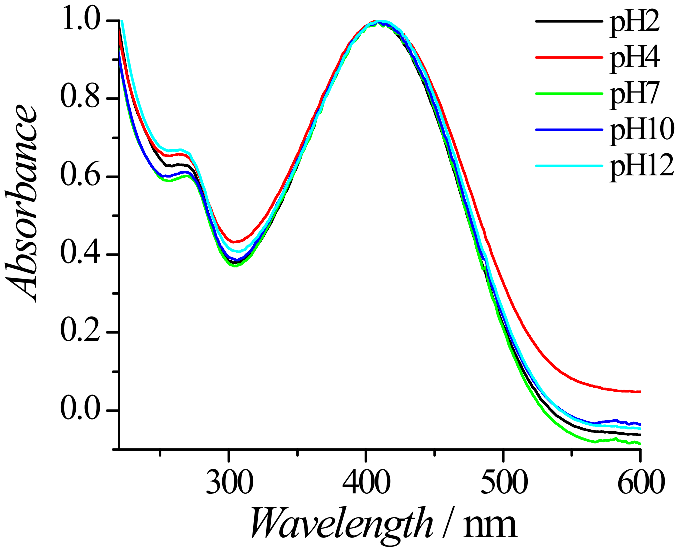

Figure S12. Normalized UV-vis spectra of pCBTh in 20 mM phosphate solution at different pH values.

[1] Kim, L. Chen, Gong, Y. Osada, *Macromolecules* **1999**, *32*, 3964-3969.

[2] X. Jiang, M. Ahmed, Z. Deng, R. Narain, *Bioconjugate Chem.* **2009**, *20*, 994-1001.

[3] D. Mawad, E. Stewart, D. L. Officer, T. Romeo, P. Wagner, K. Wagner, G. G. Wallace, *Adv. Funct. Mater.* **2012**, *22*, 2692-2699.

[4] A. E. Javier, S. N. Patel, D. T. Hallinan, V. Srinivasan, N. P. Balsara, *Angew. Chem., Int. Ed.***2011**, *50*, 9848-9851.

[5] W. S. Huang, B. D. Humphrey, A. G. Macdiarmid, *J. Chem. Soc., Faraday Trans.***1986**, *82*, 2385-2400.

[6] B. Cao, L. Li, H. Wu, Q. Tang, B. Sun, H. Dong, J. Zhe, G. Cheng, *Chem. Commun.* **2014**, *50*, 3234-3237.
